# Do Onodi Cells Influence the Onset of Sphenoiditis? A Multicentric Cross-Sectional Study

**DOI:** 10.3390/jcm14103508

**Published:** 2025-05-16

**Authors:** Gian Luca Fadda, Alberto Maria Saibene, Chiara Rustichelli, Letizia Nitro, Mario Lentini, Federica Maria Parisi, Salvatore Cocuzza, Giovanni Cavallo, Eugenio De Corso, Antonino Maniaci

**Affiliations:** 1 Department of Otolaryngology, University of Turin, “San Luigi Gonzaga” Hospital, Regione Gonzole 10, Orbassano, 10043 Turin, Italy; chiararustichelli75@gmail.com (C.R.); giovanni.cavallo@unito.it (G.C.); 2 Otolaryngology Unit, Santi Paolo e Carlo Hospital, Department of Health Sciences, Università degli Studi di Milano, 20142 Milan, Italy; alberto.saibene@gmail.com; 3 Otolaryngology Unit, ASST Santi Paolo e Carlo, Università degli Studi di Milano, 20142 Milan, Italy; letizia.nitro@gmail.com; 4 ASP Ragusa-Hospital Giovanni Paolo II, 97100 Ragusa, Italy; marlentini@tiscali.it (M.L.); antonino.maniaci@unikore.it (A.M.); 5 Department of Medical and Surgical Sciences and Advanced Technologies “GF Ingrassia”, ENT Section, University of Catania, Via S. Sofia, 78, 95125 Catania, Italy; federicamariaparisi@gmail.com (F.M.P.); s.cocuzza@unict.it (S.C.); 6 Otorhinolaryngology, “A. Gemelli” Unversitary Hospital IRCCS, 00168 Rome, Italy; eugenio.decorso@gmail.com; 7 Department of Medicine and Surgery, School of Medicine, University of Enna “Kore”, 94100 Enna, Italy

**Keywords:** anatomical landmarks, tomography, sphenoiditis, Onodi cells, paranasal sinuses

## Abstract

**Background:** Sphenoiditis poses diagnostic and treatment challenges due to its complex anatomy and potential for serious complications. Anatomic variations, such as Onodi cells, could play a role in the onset and spreading of inflammation. The diagnosis and treatment of sphenoiditis can be more difficult if Onodi cells are present, especially due to their proximity to delicate vital tissues. **Objectives**: The purpose of this study was to look at the frequency, features, and relationship between Onodi cells and sphenoiditis. **Methods**: This multicentric study comprised 550 people who received sinonasal CT imaging. The Thimmaiah classification was used to assess the presence and features of Onodi cells, and radiographic results were used to diagnose sphenoiditis. We conducted univariate and multivariate logistic regression to evaluate the relationships between sphenoiditis and Onodi cells. **Results**: The prevalence of Onodi cells was 32.40%, with a higher prevalence on the right side (18.40%) compared to the left side (8.40%). The multivariable analysis revealed a significant correlation between right-side Type II Onodi cells and a higher incidence of sphenoiditis (OR = 6.81, 95% CI: 1.14–38.97, *p* = 0.029). In the univariable analysis (OR = 3.00, 95% CI: 1.15–6.96, *p* = 0.015), but not in the multivariable analysis, the presence of Type I Onodi cells on the left side was significantly associated with sphenoiditis. **Conclusions**: There may be a link between a higher incidence of sphenoiditis and the presence of Type II Onodi cells on the right side. In order to validate these findings and clarify the underlying processes of this connection, more prospective research is required.

## 1. Introduction

Adolf Onodi (1857–1919), a rhinolaryngology pioneer, was the first to document the Onodi cell *(cellula sphenoethmoidalis)* in 1903 [1]. This anatomic variation involves the most posterior or lateral ethmoid air cell that extends superiorly and/or laterally beyond the sphenoid sinus. In endoscopic sinus surgery (ESS), these are important due to their presence in the Optic nerve canal (ONC) and Internal carotid artery (ICA), and their relationship with the pneumatization of the pterygoid recess and the attachment of sphenoidal septa. In the presence of complete pneumatization of the sphenoid sinus, these neurovascular structures may be seen projecting into the air space, which may be dehiscent and sometimes without a bony barrier [2]. Thus, preoperative assessment with paranasal sinus (PNS) computed tomography (CT) scans is mandatory to prevent iatrogenic trauma, which can cause significant complications [2,3,4,5] Sphenoiditis, which is the inflammation of the sphenoid sinus based on radiological findings including mucosal thickening > 3 mm and/or partial or complete opacification of the sphenoid sinus, is a disease entity with diagnostic and therapeutic limitations, mainly because of the complicated anatomy of the sinus and the risk of severe complications, such as intracranial spread [6,7]. In particular, eccentrically pneumatized sphenoid sinuses with inflammatory disease may complicate intracranial extension, causing headaches, double vision and cranial nerve palsies [8,9]. Fadda et al. reported an unusual case of abducens nerve palsy caused by invasive sinus actinomycosis induced by an abnormally large sphenoid sinus anatomy [10]. Cases like the present one demonstrate the relevance of knowing about the anatomy and variations in the sphenoid sinuses for successful diagnosis and treatment.

To halt the progression of the illness and avert potentially fatal outcomes, the prompt identification and treatment of sphenoiditis are essential [11]. An interesting anatomical variation in the posterior ethmoid near the sphenoid sinus is the presence of Onodi cells, which extend toward or into the sphenoid bone [5,6,7,8,9,10,11,12]. The incidence of OCs varies throughout populations, ranging from 8% to 65% [7,13,14,15,16,17,18,19,20].

These cells may have a role in the onset and progression of sphenoiditis, which makes them clinically significant [21]. Even though Onodi cells are known to be important in sphenoiditis, research on the connection between these structural differences and inflammatory disease is still underway [22]. According to certain research, there may be a connection between Onodi cells and a higher incidence of sphenoiditis [23,24]. For instance, patients with Onodi cells reported a considerably higher prevalence of sphenoiditis than those without the condition [25]. This finding may be related to decreased sphenoid sinus drainage. Onodi cells and sphenoiditis, however, have not been conclusively linked by other researchers [26,27]. According to Kim et al., there is no statistically significant difference between patients with and without Onodi cells in terms of the incidence of sphenoiditis [28].

These contradictory results emphasize the need for further investigations to fully understand the connection between sphenoiditis and Onodi cells. Furthermore, little research has been performed on the effects of particular Onodi cell subtypes, which differ in size, shape, degree of pneumatization, and protrusions, on the onset of sphenoiditis [23]. It is yet unclear what these anatomical differences mean clinically in terms of sphenoiditis [29]. The purpose of this cross-sectional study was to examine the characteristics and prevalence of Onodi cells in a group of patients, as well as any possible correlation between the diagnosis of sphenoiditis and the existence of Onodi cells, including subtypes and protrusions. Our knowledge of these structural changes and their clinical importance could be advanced by investigating the prevalence, traits, and connection between Onodi cells and sphenoiditis. The results may offer useful information to caregivers, which could help with sphenoiditis diagnosis and treatment and eventually lead to better patient outcomes.

## 2. Materials and Methods

### 2.1. Study Design and Setting

**Figure 1:**
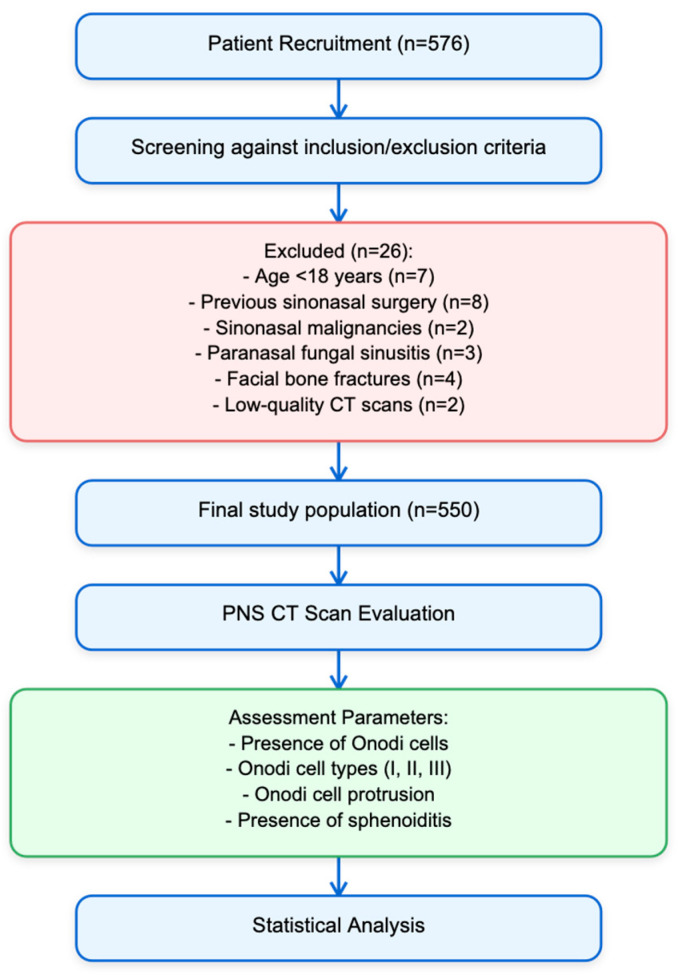
Flowchart study protocol.

On 10 April 2025, we retrieved studies describing the design, conduct, and reporting of cross-sectional clinical studies from the EQUATOR network (https://www.equator-network.org/). Further research on the guidelines’ references was performed to identify relevant publications. We then selected and adhered to the Strengthening the Reporting of Observational Studies in Epidemiology (STROBE) checklist [29]. Between November 2017 and December 2023, this cross-sectional multicentric study was carried out at “San Luigi Gonzaga” University Hospital. Participants in the study included patients who had PNS CT scans for any number of purposes. The study protocol is summarized in Figure 1.

The inclusion criteria were individuals older than 18 with rhinonasal symptoms (nasal obstruction, chronic sinusitis, rhinorrhoea) requiring radiological evaluation. Patients were recruited from otorhinolaryngology clinics at participating centers. The following were the exclusion criteria: patients under the age of eighteen, patients with a history of sinonasal surgery, patients with malignancies in the sinonasal region, paranasal fungal sinusitis, osteofribroma, fibrous dysplasia, facial bone fractures, previous head trauma and massive nasal polyposis that could modify normal anatomy, and patients with incomplete or low-quality CT scans. The main outcome variable was the existence of sphenoiditis. Sphenoiditis was diagnosed based on radiological findings including mucosal thickening > 3 mm, the partial or complete opacification of the sphenoid sinus, or the presence of air–fluid levels within the sphenoid sinus. The absence of sphenoiditis was determined by a normal mucosal thickness (<3 mm), the absence of opacification, and the absence of air–fluid levels within the sphenoid sinus.

**Figure 2:**
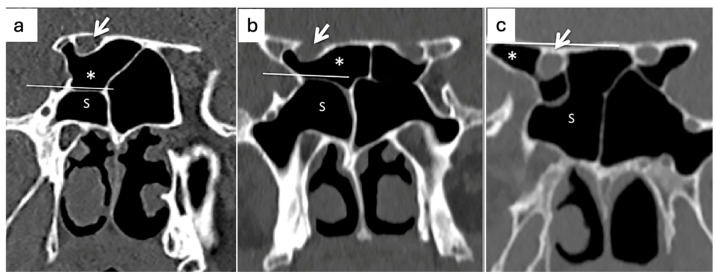
Coronal PNS CT scans. (**a**) Onodi cell (*) type I pattern of pneumatization is above and medial the horizontal line. (**b**) Onodi cell (*) type II is above and below the horizontal line. (**c**) Onodi cell (*) type III is below the horizontal line. A horizontal line was drawn at the uppermost part of sphenoid sinus (s) in the coronal image. Optic canals (white arrows) with bulging > 5 mm (type 4) are shown [30,31].

All CT scans were reviewed on the axial, coronal, and sagittal planes to better identify the presence of Onodi cells, as described in the Onodi and Fadda study [14]. The main exposure variable was the existence of Onodi cells, which were detected and categorized using the Thimmaiah et al. categorization system [30] depending on their type (Type I, II, or III). Three patterns of Onodi Cell pneumatization were assessed: Type I (superior) if the sphenoethmoidal cell was located above and medial to the sphenoid sinus; Type II (superolateral) if the air cell was located both above and below to a horizontal line drawn at the uppermost part of SS in a coronal plane; and Type III if the sphenoethmoidal cell was located below the horizontal line (Figure 2).

Additionally, Onodi cell protrusion was measured and classified as bilateral, left-sided, or right-sided. Medical records of the subjects were consulted to gather demographic information, such as age and sex. Two impartial radiologists who were blind to the clinical details of the individuals evaluated the PNS CT scans. Any differences in the radiological evaluations were settled by consensus. The Institutional Review Board (IRB) of “San Luigi Gonzaga” University Hospital accepted the study protocol (CE 44/2022). Written informed consent was obtained from each participant before they could be included in the study.

### 2.2. Statistical Analysis

Frequencies and percentages were used to represent categorical variables, while means, standard deviations, medians, and interquartile ranges, depending on the distribution of the continuous data, were used to portray them. When comparing categorical variables between groups, the chi-square test or Fisher’s exact test was employed, and when comparing continuous variables, the Student’s *t*-test or Mann–Whitney U test was used, as appropriate. On 31 March 2025, the OpenEpi software (Open-Source Epidemiologic Statistics for Public Health, Version 3.01, www.OpenEpi.com) was used to compute the sample size. Given a 25% prevalence of Onodi cells [14] and a 15% prevalence of sphenoiditis [11], together with an 80% power and a 5% significance level, the bare minimum sample size needed was calculated to be 500 participants. The study included both univariate and multivariate logistic regression analysis to evaluate the relationships between the diagnosis of sphenoiditis and the presence and features of Onodi cells. We computed odds ratios (ORs) and the 95% confidence intervals (CIs) that go along with them. In the multivariable model, variables that had a *p*-value of less than 0.1 in the univariable analysis were included. *p*-values less than 0.05 were deemed statistically significant. The R program (Version 4.0.3, R Foundation for Statistical Computing, Vienna, Austria) and IBM SPSS Statistics for Windows (Version 29.0, IBM Corp., Armonk, NY, USA) were used for all statistical analyses.

## 3. Results

### 3.1. Demographic Characteristics

The study included a total of 550 participants, comprising 237 (43.10%) females and 313 (56.90%) males (Table 1). The sex distribution did not differ significantly between the groups (*p* = 0.089). Of the 550 participants, 372 (67.60%) had no identifiable Onodi cells (OCs), while 178 (32,4%) presented OCs. Among those with OCs, 101 (18.40%) had right-sided OCs, 46 (8.40%) had left-sided OCs, and 31 (5.60%) had bilateral OCs. The differences in the distribution of OCs were statistically significant (*p* < 0.001). A further analysis of the OC types revealed that Type I OCs were present in 47 (8.50%) participants on the right side and 18 (3.30%) on the left side, with 485 (88.20%) participants having no Type I OCs (*p* < 0.001). Type II OCs were present in 35 (6.40%) participants on the right side and 4 (0.70%) on the left side, with 511 (92.90%) participants having no Type II OCs (*p* < 0.001). Type III OCs were present in 22 (4.00%) participants on the right side and 2 (0.40%) on the left side, with 526 (95.60%) participants having no Type III OCs (*p* < 0.001). Regarding ONC protrusion, 18 (3.30%) participants had right-sided protrusion, 3 (0.50%) had left-sided protrusion, and 3 (0.50%) had bilateral protrusion. The remaining 529 (96.20%) participants did not exhibit any Onodi cell protrusion. The differences in protrusion patterns were statistically significant (*p* < 0.001).

### 3.2. Onodi Cells and Sphenoiditis

The relationship between the presence and characteristics of Onodi cells and sphenoiditis was further analyzed (Table 2). The distribution of sphenoiditis based on the presence of Onodi cells did not differ significantly (*p* = 0.344). Regardless of the laterality of Onodi cells, the majority of participants (80–90%) did not have sphenoiditis. The distribution of sphenoiditis was also not significantly associated with the different types of Onodi cells. For Type I OCs (*p* = 0.069), Type II OCs (*p* = 0.705), and Type III OCs (*p* = 0.869), the majority of participants (78–88%) did not have sphenoiditis. Similarly, the distribution of sphenoiditis did not differ significantly based on Onodi cell protrusion (*p* = 0.272). The majority of participants (83–87%) without protrusion or with unilateral protrusion did not have sphenoiditis.

### 3.3. Predictors Analysis

**Figure 3:**
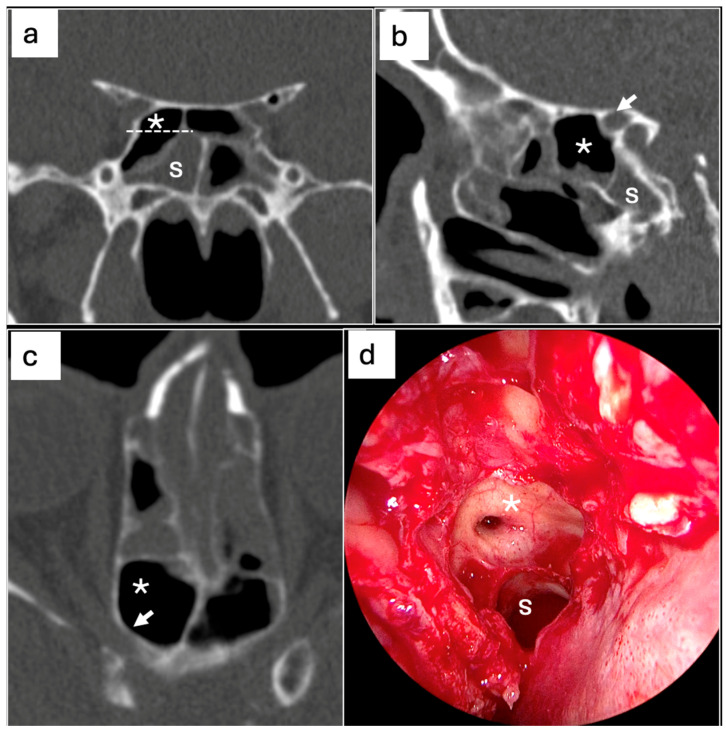
PNS CT imaging showing correlation between Type II Onodi cell and right-sided sphenoiditis. (**a**) Coronal, (**b**) sagittal and (**c**) axial PNS CT scan showing type II Onodi cell (asterisk) extending superolaterally from the sphenoid sinus, and sphenoiditis with complete opacification of the right sphenoid sinus (s) and optic canals (white arrow); (**d**) endoscopic right sphenoid sinus (s) and Onodi cell (asterisk) are shown.

**Figure 4:**
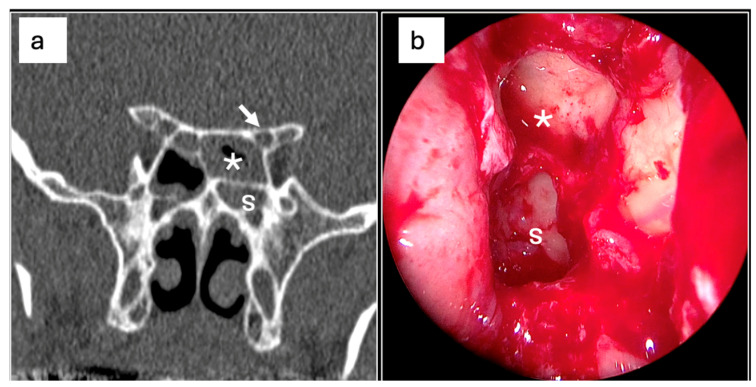
PNS CT and endoscopic images showing correlation between Type II Onodi cell and left-sided sphenoiditis. (**a**) Coronal PNS CT images showing Type II Onodi cell (asterisk) with superolateral extension and a complete opacification of the left sphenoid sinus. Note the proximity to the optic canal (white arrow), highlighting the clinical significance of this anatomical variation. (**b**) Endoscopic view of the left sphenoid sinus (s), showing the inflamed Onodi cell (asterisk) with purulent discharge visible at the ostium.

The univariable and multivariable logistic regression analyses were performed to assess the associations between the presence and characteristics of Onodi cells and the diagnosis of sphenoiditis (Table 3). In the univariable analysis for right sphenoiditis, the presence of Type II Onodi cells was not significantly associated with the diagnosis of sphenoiditis (OR = 2.37, 95% CI: 0.66–6.62, *p* = 0.132) (Figure 3). However, in the multivariable analysis, the presence of Type II Onodi cells was significantly associated with an increased risk of sphenoiditis (OR = 6.81, 95% CI: 1.14–38.97, *p* = 0.029). Conversely, for left-sided sphenoiditis, the presence of Onodi cells was significantly associated with an increased risk sphenoiditis in the univariable analysis (OR = 2.25, 95% CI: 0.97–4.75, *p* = 0.044) (Figure 4). However, this association was not significant in the multivariable analysis (OR = 1.33, 95% CI: 0.31–6.46, *p* = 0.712). Moreover, the presence of Type I Onodi cells was significantly associated with increased sensitivity for the detection of sphenoiditis (OR = 3.00, 95% CI: 1.15–6.96, *p* = 0.015). However, this association was not significant in the multivariable analysis (OR = 2.98, 95% CI: 0.57–12.21, *p* = 0.157).

## 4. Discussion

Onodi cells are the most posterior ethmoid air cells that pneumatize superolateral, superior, or lateral to the sphenoid sinus and surround the optic canal; also, they can penetrate into the anterior clinoid process [32]. For the first time, we examined the frequency, features, and possible correlation between Onodi cell types and sphenoiditis in this cross-sectional investigation. According to our research, having Onodi cells—especially Type II Onodi cells on the right side—may raise your risk of developing sphenoiditis.

The incidence of OC varies significantly across previous studies. In studies based solely on axial projection PNS CT imaging, the reported incidence of Onodi cells is generally low (8–24%) [33,34]. In this regard, we consider it mandatory that the radiological identification and analysis of OC be conducted in all three spatial planes (axial, coronal, and sagittal). In a detailed study based on sagittal CT imaging, Tomovic et al. [7] reported an OC incidence of 65.3%; similarly, Wada K et al. [16] reported an incidence of 50.8%.

In our study population, the prevalence of Onodi cells was 32.40% (178/550 PNS CT scan), which is in line with the 8–65% range noted in the literature [2,7,13,15,16,17,18,19,20]. Liu J et al. [20] reported 33 Onodi cells (21.57%) on 153 PNS CT scans. Kang YJ et al. [35] reported Onodi cells in 449 patients (51.2%), and of these, 301 (34.4%) had bilateral Onodi cells. Asian studies have reported incidences of Onodi cells of 32.7–60% [12,36].

According to earlier research [7,23], the right side has a higher prevalence of Onodi cells (18.40%) than the left (8.40%). Genetic, environmental, and developmental variables may account for the variance in Onodi cell prevalence among various groups [13]. In the multivariable analysis, we discovered that Type II Onodi cells on the right side were significantly associated with an increased risk of sphenoiditis, even though the overall presence of Onodi cells was not significantly associated with sphenoiditis (*p* = 0.344). (OR = 6.81, 95% CI: 1.14–38.97, *p* = 0.029). This result is consistent with research by Lee et al. [1,2,3,4,5,6], which found that individuals with Onodi cells had a higher prevalence of sphenoiditis. The altered drainage channel and greater likelihood of obstruction in the sphenoid sinus may be the cause of the increased risk of sphenoiditis in the presence of Type II Onodi cells [21,22].

In a patient with sudden vision loss, complicated sphenoid sinusitis should always be suspected, in addition to the possible secondary involvement of Onodi cell mucocele. In this regard, AA [37,38,39] reported a case with a history of previous sinus surgery, and visited the emergency department due to abrupt ocular pain following visual loss where an Onodi cell mucocele with bone dehiscence in the orbital apex was seen on CT; this required emergency endoscopic surgery.

Geng C et al. [39] described eight patients with Onodi cell mucocele, and cholesterol granuloma in four of them. They reported nasal symptoms and ocular symptoms, and all patients underwent endoscopic sinus surgery, after which significant improvement was seen.

Intriguingly, in the univariable analysis, the left side’s Type I Onodi cell presence was significantly linked to a higher sensitivity for the detection of sphenoiditis (OR = 3.00, 95% CI: 1.15–6.96, *p* = 0.015). In the multivariable analysis, however, this connection was not significant (OR = 2.98, 95% CI: 0.57–12.21, *p* = 0.157). This finding has unknown clinical implications, which calls for more research. Tan et al.’s [26] results, which showed no discernible effect of ONC on sinus surgery outcomes, are in line with our study’s lack of a significant correlation between Onodi cell protrusion and sphenoiditis (*p* = 0.272). Nevertheless, Onodi cell protrusion may still have an impact on surgical planning and the avoidance of problems [40,41].

Although there is no effective classification method for the proper opening of the sphenoid sinus, in agreement with Wada K et al. [16], we believe that often the ostium of the sphenoid sinus cannot be identified without resecting the inferior part of the superior turbinate. In fact, the ostium of the sphenoid sinus is generally situated in one third of the superior turbinate [28], and the vertical distance between the sphenoid ostium and the choana is an important surgical measure in determining the entry point to the sphenoid [18]. Doubi et al. [19] found that the presence of OC lengthens this line, making the ostium higher with regard to the choana.

If the sinus ostium is missed during surgery, complications may arise; these include a cerebrospinal leak superiorly, an arterial bleed inferiorly, or a septal perforation medially.

The diseased Onodi cells are known to increase the risk of visual loss, as described by Huang EI et al. [18]. Consistent with the authors and by interpreting our findings, we may suggest the preoperative and intraoperative localization of a sphenoethmoidal cell, along with its use in the case of an intraoperative navigation during ESS to minimize potential complications.

The adoption of a defined classification system for Onodi cells [30], the high sample size (*n* = 550), and the adherence to the STROBE principles for publishing observational studies [29] are some of the study’s strengths. Our study does, however, have some shortcomings. It is not possible to establish a causal link between Onodi cells and sphenoiditis because of the cross-sectional design. Our study supports what was reported by Senturk M et al. [42], who found that the co-existence of OC ipsilaterally increased the identification of sphenoiditis 1.5-fold, and that this finding was statistically significant (*p* < 0.05). Furthermore, the study cohort may not be representative of the general population because it was restricted to patients who had PNS CT imaging for a variety of reasons, as described in the inclusion criteria, with the exclusion of patients with mucoceles of the posterior sinus compartment.

## 5. Conclusions

The aim of our work is to underline the importance of Onodi cells, because to prevent complications, it is important to be able to recognize them with CT and ESS. According to our research, a higher incidence of sphenoiditis may be linked to the presence of right-sided Type II Onodi cells. This result emphasizes the significance of determining the existence and features of Onodi cells in patients with suspected sphenoiditis. This is corroborated by the contingency analysis and the heatmap’s visual representation. Our findings need to be confirmed by bigger prospective studies using uniform diagnostic criteria to clarify the underlying mechanisms of this connection.

## Figures and Tables

**Table 1:** Demographic features of patients enrolled. Abbreviations: OC, Onodi cell; ONC, optic nerve canal; y, year.

	N° (%)	*p*-Value
Age	52, 9 ± 18 y	
Sex	550 (100%)	
Female	237 (43.1%)	
Male	313 (56.9%)	
OC	178 (32.4%)	
No	372 (67.6%)	<0.001
Right	101 (18.4%)	
Left	46 (8.4%)	
Bilateral	31 (5.6%)	
OC type I	65 (11.8%)	
No	485 (88.2%)	<0.001
Right	47 (8.5%)	
Left	18 (3.3%)	
OC type II	39 (7.1%)	
No	511 (92.9%)	<0.001
Right	35 (6.4%)	
Left	4 (0.7%)	
OC type III	24 (4.4%)	
No	526 (95.6%)	<0.001
Right	22 (4%)	
Left	2 (0.4%)	
ONC protrusion	24 (4.3%)	
No	529 (96.2%)	<0.001
Right	18 (3.3%)	
Left	3 (0.5%)	
Bilateral Protrusion	3 (0.5%)	
Sphenoiditis	70 (12.7%)	
No	480 (87.3%)	<0.001
Right	25 (4.5%)	
Left	26 (4.7%)	
Bilateral	19 (3.5%)	

**Table 2:** Contingency analysis for sphenoiditis occurrence and Onodi cells. Abbreviations: OC, Onodi cell. ONC, optic nerve canal.

	Sphenoiditis						
		No (%)	Right	Left	Bilateral	Total	*p*-Value
OC	No	328 (88.2%)	15 (4.0%)	19 (5.1%)	10 (2.7%)	372	0.344
	Right	87 (86.1%)	4 (4%)	6 (5.9%)	4 (4%)	101	
	Left	37 (80.4%)	5 (10.9%)	1 (2.2%)	3 (6.5%)	46	
	Bilateral	28 (90.3%)	1 (3.2%)	0	2 (6.5%)	31	
OC type I	No	427 (88%)	20 (4.1%)	24 (4.9%)	14 (3%)	485	0.069
	Right	37 (79%)	3 (6.3%)	2 (4.2%)	5 (10.5%)	47	
	Left	16 (88.9%)	2 (11.1%)	0	0	18	
OC type II	No	448 (87.7%)	23 (4.5%)	24 (4.7%)	16 (3.1)	511	0.705
	Right	28 (80%)	2 (5.7%)	2 (5.7%)	3 (8.6%)	35	
	Left	4 (100%)	0	0	0	4	
OC type III	No	459 (87.3%)	23 (4.4%)	26 (4.9%)	18 (3.4%)	526	0.869
	Right	19 (86.4%)	2 (9.1%)	0	1 (4.5%)	22	
	Left	2 (100%)	0	0	0	2	
ONC protrusion	No	462 (87%)	25 (4.7%)	23 (4.3%)	19 (4%)	529	0.272
	Right	15 (83.3%)	0	3 (16.7%)	0	18	
	Left	3 (100%)	0	0	0	3	
ONC bilateral protrusion	Yes	477 (87.2%)	25 (4.6%)	26 (4.7%)	19 (3.5%)	547	0.932
	No	3	0	0	0	3	

**Table 3:** Univariate and multivariate analysis assessing risk of sphenoiditis depending on variable presence. Abbreviations: OC, Onodi cell; ONC, optic nerve canal.

Right-Side Sphenoiditis		No	Yes	OR (Univariable)	OR (Multivariable)	Left-Side Sphenoiditis		No	Yes	OR (Univariable)	OR (Multivariable)
Sex	Female	217 (91.6%)	20 (8.4%)	-	-	Sex	Female	219 (92.4%)	18 (7.6%)	-	-
	Male	288 (92%)	25 (8%)	0.94 (0.51–1.76, *p* = 0.848)	0.93 (0.50–1.74, *p* = 0.808)		Male	287 (91.7%)	26 (8.3%)	1.10 (0.59–2.09, *p* = 0.761)	1.00 (0.53–1.92, *p* = 0.999)
OC	No	449 (91.8%)	40 (8.2%)	-	-	OC	No	454 (92.8%)	35 (7.2%)	-	-
	Yes	56 (91.8%)	5 (8.2%)	1.00 (0.34–2.43, *p* = 0.996)	0.32 (0.06–1.70, *p* = 0.186)		Yes	52 (85.2%)	9 (14.8%)	2.25 (0.97–4.75, *p* = 0.044)	1.33 (0.31–6.46, *p* = 0.712)
Bilateral OC	No	476 (91.7%)	43 (8.3%)	-	-	Bilateral OC	No	478 (92.1%)	41 (7.9%)	-	-
	Yes	29 (93.5%)	2 (6.5%)	0.76 (0.12–2.66, *p* = 0.718)	0.30 (0.03–1.82, *p* = 0.221)		Yes	28 (90.3%)	3 (9.7%)	1.25 (0.29–3.72, *p* = 0.724)	0.27 (0.04–1.41, *p* = 0.145)
OC type I	No	463 (91.9%)	41 (8.1%)	-	-	OC type I	No	476 (92.8%)	37 (7.2%)	-	-
	Yes	42 (91.3%)	4 (8.7%)	1.08 (0.31–2.83, *p* = 0.894)	3.73 (0.57–19.35, *p* = 0.134)		Yes	30 (81.1%)	7 (18.9%)	3.00 (1.15–6.96, *p* = 0.015)	2.98 (0.57–12.21, *p* = 0.157)
OC type II	No	485 (92.2%)	41 (7.8%)	-	-	OC type II	No	489 (92.1%)	42 (7.9%)	-	-
	Yes	20 (83.3%)	4 (16.7%)	2.37 (0.66–6.62, *p* = 0.132)	6.81 (1.14–38.97, *p* = 0.029)		Yes	17 (89.5%)	2 (10.5%)	1.37 (0.21–5.00, *p* = 0.681)	1.49 (0.18–7.25, *p* = 0.661)
OC type III	No	497 (91.7%)	45 (8.3%)	-	-	OC type III	No	490 (92.1%)	42 (7.9%)	-	-
	Yes	8 (100%)	0 (0%)	0.00 (NA, *p* = 0.987)	0.00 (NA, *p* = 0.986)		Yes	16 (88.9%)	2 (11.1%)	1.46 (0.23–5.36, *p* = 0.623)	1.40 (0.15–8.45, *p* = 0.739)
ONC protrusion	No	495 (91.8%)	44 (8.2%)	-	-	ONC protrusion	No	493 (91.8%)	44 (8.2%)	-	-
	Yes	10 (90.9%)	1 (9.1%)	1.12 (0.06–6.08, *p* = 0.912)	1.50 (0.07–10.67, *p* = 0.725)		Yes	13 (100%)	0 (0%)	0.00 (NA, *p* = 0.983)	0.00 (NA, *p* = 0.988)

## Data Availability

The original contributions presented in this study are included in the article. Further inquiries can be directed to the corresponding author.

## Abbreviations

The following abbreviations are used in this manuscript:

OC	Onodi Cell
CT	Computed Tomography
OR	Odd Ratio
ONC	Optic Nerve Canal
ICA	Internal carotid artery
ESS	Endoscopic sinus surgery
PNS	Paranasal sinus
